# Mutations in Cancer Cause Gain of Cysteine, Histidine, and Tryptophan at the Expense of a Net Loss of Arginine on the Proteome Level

**DOI:** 10.3390/biom7030049

**Published:** 2017-07-03

**Authors:** Viktoriia Tsuber, Yunus Kadamov, Lars Brautigam, Ulrika Warpman Berglund, Thomas Helleday

**Affiliations:** 1 Science for Life Laboratory, Division of Translational Medicine and Chemical Biology, Department of Medical Biochemistry and Biophysics, Karolinska Institutet, Stockholm S171 21, Sweden; lars.brautigam@scilifelab.se (L.B.); ulrika.warpmanberglund@scilifelab.se (U.W.B.); 2 Department of Medical, Bioorganic and Biological Chemistry, Ukrainian Medical Stomatological Academy, Poltava 36011, Ukraine; yunik2932@gmail.com

**Keywords:** mutations in cancer, amino acid substitutions, somatic evolution, arginine

## Abstract

Accumulation of somatic mutations is critical for the transition of a normal cell to become cancerous. Mutations cause amino acid substitutions that change properties of proteins. However, it has not been studied as to what extent the composition and accordingly chemical properties of the cell proteome is altered as a result of the increased mutation load in cancer. Here, we analyzed data on amino acid substitutions caused by mutations in about 2000 protein coding genes from the Cancer Cell Line Encyclopedia that contains information on nucleotide and amino acid alterations in 782 cancer cell lines, and validated the analysis with information on amino acid substitutions for the same set of proteins in the Catalogue of Somatic Mutations in Cancer (COSMIC; v78) in circa 18,000 tumor samples. We found that nonsynonymous single nucleotide substitutions in the analyzed proteome subset ultimately result in a net gain of cysteine, histidine, and tryptophan at the expense of a net loss of arginine. The extraordinary loss of arginine may be attributed to some extent to composition of its codons as well as to the importance of arginine in the functioning of prominent tumor suppressor proteins like p53.

## 1. Introduction

Increased mutagenesis is a hallmark of cancer. Many human tumors harbor multiple mutations. Some of cancer mutations are “driver” mutations that activate oncogenes or disable tumor suppressor genes. They are positively selected in cancer since they promote cancer growth. However, most of the mutations in the cancer cell are “passenger” mutations that do not contribute to cancer progression.

Most of the current research concentrates on finding algorithms able to distinguish the driver and passenger mutations as well as on the effects of driver mutations on cancer initiation and progression. Mutation patterns and mutation signatures in different cancers have been studied in detail on the nucleotide level [1,2] and on the amino acid level [3]. However, little is known about the general mutational landscape on the proteome level, i.e., how much the somatic evolution in cancer changes the amino acid composition of the cell proteins and accordingly the chemical properties of the proteome.

In the present study, we analyzed data on amino acid substitutions in mutations in a proteome subset of circa 2000 proteins from the Cancer Cell Line Encyclopedia (CCLE) [4] that contains consistent information on genomic and proteomic alterations in 782 cancer cell lines. The analysis was validated and expanded with information on amino acid substitutions for the same set of proteins in COSMIC (v78), the Catalogue Of Somatic Mutations in Cancer [5] in circa 18,000 tumor samples.

Here, we demonstrate that the total outcome of all nonsynonymous single nucleotide substitutions in approximately two thousand proteins in more than 18,000 cancer samples is a gain of cysteine, histidine, and tryptophan at the expense of a net loss of arginine. The exceptionally high number of substitutions of arginine in cancer can be associated with an unusually high mutation rate in four of the six codons that code for the amino acid as well as with targeted loss of arginine in several important tumor suppressor proteins that are often mutated in cancer. Additionally, the marked gain of cysteine, histidine, and tryptophan may increase the total antioxidant and metal-binding capacity of the proteome of the cancer cell and thus potentially provide a nonspecific compensatory mechanism to alleviate consequences of the cancer-related aggravation of oxidative stress. Furthermore, these results suggest that an increased demand for cysteine, histidine, or tryptophan for protein synthesis in the cancer cell may place additional pressure on its metabolism and may make some cancers sensitive to deprivation of the amino acids.

## 2. Results

We analyzed data on amino acid substitutions in the CCLE database that result from nonsynonymous single nucleotide substitutions in genes that code for 2164 proteins. The analyzed proteome subset comprises about one tenth of the human protein-encoding genes. The genes in the CCLE database have been consistently studied in 782 cancer cell lines that belong to 23 tissue types in the human organism. We further validated the results with an analysis of amino acid substitutions in the same set of proteins in the COSMIC database in circa 18,000 tumor samples.

### 2.1. C>T and G>A Changes Are the Most Numerous Nucleotide Transformations

**Figure 1:**
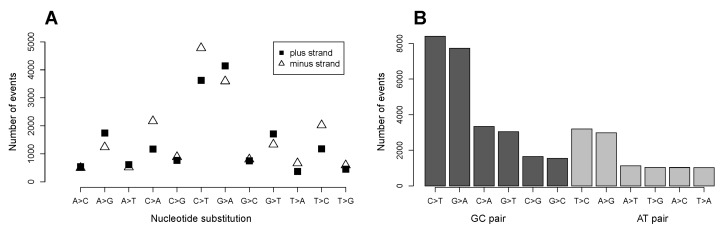
Single nucleotide substitutions in the Cancer Cell Line Encyclopedia (CCLE) database. (**A**) Nucleotide substitutions in the coding and noncoding strands; circa 17,000 substitutions on the coding strand and ca. 19,000 substitutions on the noncoding strand; and (**B**) Difference in numbers of mutations between the GC and AT pairs of nucleotides. The graph comprises the data from the both coding and noncoding strands.

We analyzed data on single nucleotide substitutions that cause missense (circa 36,000 events) and nonsense mutations (ca. 3000 events) in the CCLE database. The numbers do not include splice mutations that we excluded from the analysis. Double nucleotide substitutions were only responsible for 1.3% of all non-splice mutations in the database and were not included in our analysis. C>T and its counterpart G>A changes are the most numerous nucleotide substitutions on the both coding and noncoding strands (Figure 1A). The C>T transition corresponds to the high deamination ability of cytosine, and this mutation is particularly found in CpG rich sequences where a methylated cytosine is by deamination converted directly to thymine [6]. The G>A change is explained by oxidation of guanine [7] and is in an agreement with existing data [1,2]. When the data from both strands is considered, the GC pair of nucleotides is mutated in 71.2% of all observations, and the AT pair is mutated in 28.8% (Figure 1B).

### 2.2. The Number of Coding Transformations of Amino Acids Is Not Proportional to Their Content in Proteins. Arginine Is Most Affected by Mutations Despite Not Being Most Abundant

**Figure 2:**
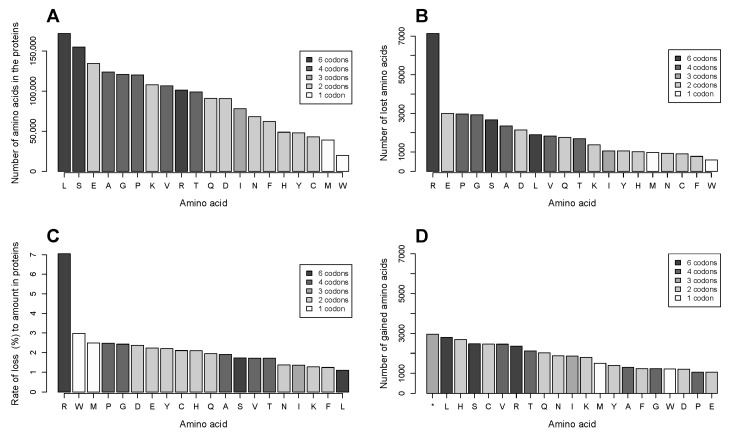
Amino acid composition of the analyzed set of proteins and substitutions in the proteins in the CCLE database. (**A**) Amino acid content in the 2164 proteins. In this and the following graphs, the bars are colored by the number of codons that code for each amino acid: amino acids are denoted with a one-letter code, and truncation is denoted as “*”. (**B**) Frequencies of loss of amino acids due to mutations in the analyzed proteome subset in the 782 cancer cell lines. (**C**) Rate of loss for each amino acid. It is calculated as the ratio of the number of the substitution events for an amino acid in the CCLE database to its amount in the 2164 proteins. (**D**) Frequencies of gain of amino acids due to mutations in the analyzed proteome subset in the 782 cancer cell lines.

We analyzed effects of single nucleotide substitutions in a proteome subset that consists of 2164 proteins. The protein length ranges from 54 amino acids (phorbol-12-myristate-13-acetate-induced protein 1) to 7968 (obscurin), with an outlier titin (34,350 amino acids). The median length is 604 amino acids; the mean length (titin excluded) is 830 amino acids. The total number of all nucleotide substitutions that bring about the missense and nonsense mutations is circa 36,000. Leucine (L) and serine (S) are the most abundant amino acids; methionine (M) and tryptophan (W) are the rarest (Figure 2A).

As a result of a nonsynonymous missense mutation, an amino acid is lost and another is incorporated into the protein in its place, i.e., gained as a result of the mutation. Similarly, an amino acid is lost in a nonsense mutation, resulting in a truncated protein. In the analyzed data, arginine (R) is by far most frequently lost due to mutations (Figure 2B). The preferential loss of arginine is in agreement with data obtained in an analysis of mutations listed in the COSMIC database [3].

We calculated the rate of loss for each amino acid as a ratio of the number of events of its loss to its amount in the proteins under study (Figure 2C). The rate of loss was significantly different for the amino acids (chi-squared = 13,093; *p* < 2.2 × 10^−16^). Arginine is the only amino acid with the rate of loss that differs significantly in the chi-square test from all other amino acids. The *p* value of the difference in the loss rate between arginine and next in line tryptophan is 3.46 × 10^−90^ (pairwise comparisons, Bonferroni correction for multiple testing). The most abundant amino acid leucine demonstrates the smallest rate of loss among all the amino acids.

### 2.3. Gains of Histidine, Cysteine, and Tryptophan and Loss of Arginine Are Characteristic for the Total Outcome of All Coding Mutations in the Proteome

Our analysis of frequencies of gain of each amino acid in the data showed that the most abundant gain is truncation of proteins due to introduction of nonsense codons by mutations, closely followed by high numbers of leucine, histidine (H), serine, cysteine (C), and valine (V) obtained in the substitutions (Figure 2D).

**Figure 3:**
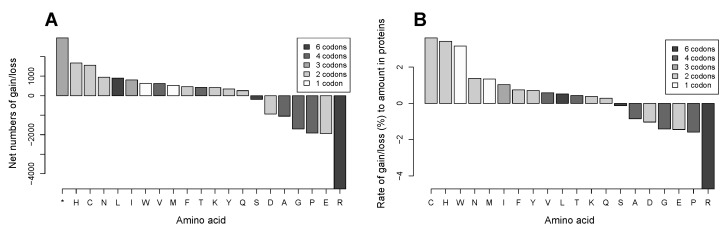
Gain and loss of amino acids in substitutions in the CCLE database. (**A**) Net frequencies of gain/loss of amino acids in the analyzed proteome subset in the 782 cancer cell lines; and (**B**) Rate of gain/loss for each amino acid as the ratio of the net frequencies of its gain/loss to its amount in the 2164 proteins.

In order to see how much the mutations change general properties, i.e., acidity/basicity, hydrophobicity, etc. of the cancer cell proteome, we calculated the net change in the amino acid substitution frequencies by subtracting the frequency of the gain of an amino acid from the frequency of its loss. Apart from the truncation, the most numerous net gains are histidine and cysteine, the most prominent loss is arginine, followed by threefold smaller losses of glutamate (E) and proline (P) (Figure 3A). Other substitutions only change the net content of the other amino acids in the proteome to a very small extent, though they probably alter properties of individual proteins.

Amino acids are found in different quantities in the proteome depending on their functions. For instance, the most abundant amino acid leucine is 8.6 times more numerous than the least abundant tryptophan, 4.4 times more numerous than methionine, and 4 times more numerous than cysteine in the analyzed set of proteins (Figure 2A). It can be assumed that a net gain of 100 residues of tryptophan or methionine may has a greater effect on general chemical properties of the proteome than the same net gain of leucine. We calculated rates of gain or loss for each amino acid as ratios of its net gain/loss frequency to the total amount of the amino acid in the proteins (Figure 3B). Significant differences between amino acids were found in the chi-square test both in the rate of net loss (chi-squared = 9243; *p* < 2.2 × 10^−16^) and in the rate of net gain (chi-squared = 8730; *p* < 2.2 × 10^−16^).

We found that the rates of gain are highest for cysteine, histidine, and tryptophan, and that they are significantly different from the next in line glutamine (N) (*p* value of the difference between cysteine and glutamine is 7.63 × 10^−123^ (pairwise comparisons in chi-square test, Bonferroni correction for multiple testing), while there is no significant difference between cysteine and histidine or cysteine and tryptophan. The rate of loss is by far most extraordinary for arginine, being threefold higher than that for the closest amino acid proline and 36 times higher than that for the least lost serine. On the proteome level, the effect may be significantly amplified because many proteins are present in multiple copies in the cell. Thus, the outcome of amino acid substitutions on the proteome level is a gain of cysteine, histidine, and tryptophan accompanied by a net loss of arginine. The change in amino acid composition may decrease basicity of the cancer cell proteome. Whereas both arginine and histidine have basic side chains, the pKa of the side chain of arginine is 12.48, while that of histidine is 6.04. Furthermore, the decrease in basicity is exacerbated by the net loss of arginine, being much bigger than the net gain of histidine. Besides, chemical properties and functions of the gained amino acids suggest that the changes may increase the total antioxidant and metal binding capacity of the cancer cell proteome. The free thiol group of cysteine is a strong reducing agent that is mostly responsible for nonspecific antioxidant properties of cellular proteins. Additionally, both histidine and cysteine can bind metal ions with high affinity. In proteins, they can bind free redox active iron atoms thus preventing them from participation in the Fenton reaction that propagates oxidation damage. Also, an introduction of histidine residues may potentially create new catalytic sites in proteins as histidine serves as a proton shuttle in catalytic reactions in the cell.

### 2.4. Changes of Arg>Cys, Arg>His, Arg>Gln, Arg>Trp, Glu>Lys and Pro>Leu Are the Few High Frequency Substitutions among 160 Possible Transformations for All Amino Acids in Single Nucleotide Substitutions in the Analyzed Proteome Subset

**Figure 4:**
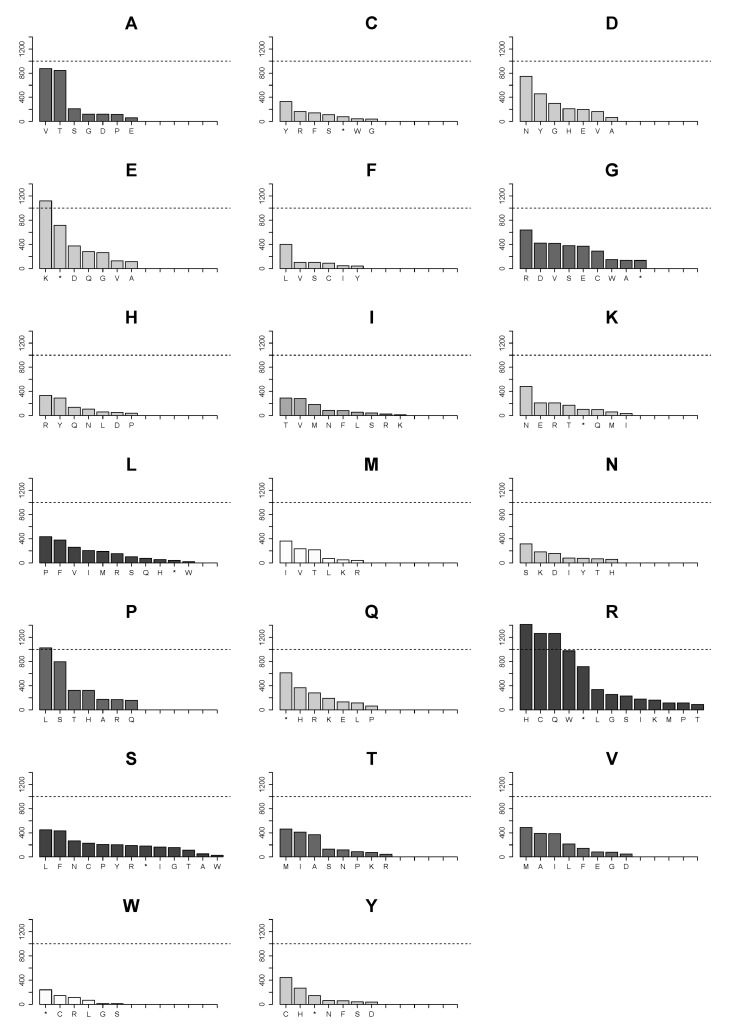
Landscape of ways of loss of each amino acid in individual substitutions in mutations in the CCLE database. The dotted horizontal line marks 1000 substitution events. The bars in each graph are colored according to the number of codons for a respective amino acid similarly to the previous graphs. The letter in the title of each graph denotes the amino acid that is lost in mutations.

**Figure 5:**
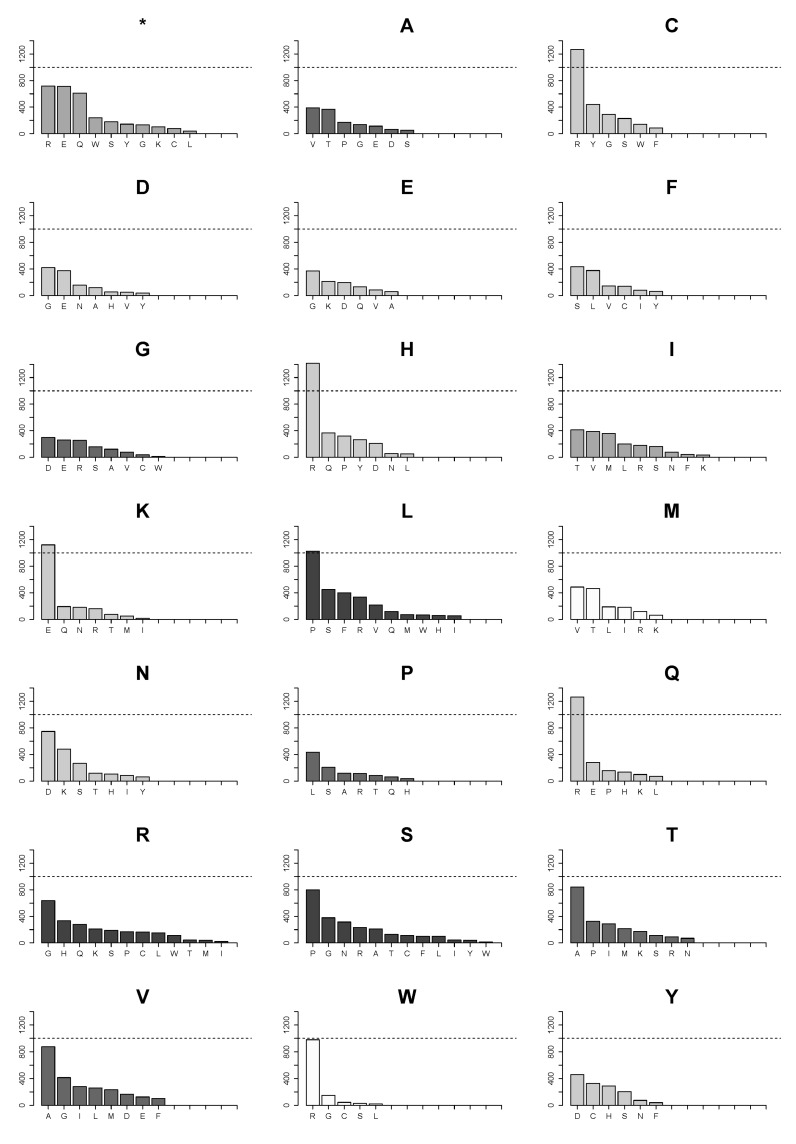
Landscape of ways of gain of each amino acid in individual substitutions that result in introductions of each of 20 amino acids and a truncation of proteins in mutations in the CCLE database. The dotted horizontal line marks 1000 substitution events. The bars in each graph are colored according to the number of codons for a respective amino acid similarly to the previous graphs. The letter in the title of each graph denotes an amino acid that is gained in mutations.

We further examined substitution patterns of individual amino acids. Figure 4 presents all possible ways of loss for each amino acid due to single nucleotide substitutions in the CCLE database. Likewise, Figure 5 illustrates all possible ways of how an amino acid can be introduced in substitutions. The two graphs recapitulate all frequencies of both gain and loss of individual amino acids in the CCLE database. The data suggest that for each amino acid, the numbers of other individual amino acids it can be substituted with (Figure 4) and the numbers of amino acids that give rise to it in substitutions (Figure 5) are related to the number of codons, with serine, leucine, and arginine having the highest number in both cases.

However, frequencies of individual substitutions vary considerably. Among circa 36,000 substitution events, very few types of amino acid substitutions reached an arbitrary cutoff of 1000 (Figure 4) in the analyzed set of proteins in the 782 cancer cell lines in the CCLE database. The only prominent substitutions apart from the substitutions of arginine are glutamate being replaced with lysine and proline with leucine. However, substitutions of arginine with cysteine, histidine, and glutamine considerably exceed 1000 and a substitution of arginine with tryptophan is just below the number. Beside the more modest numbers of substitution events for the rest of amino acids, they are more evenly distributed in each case. For instance, serine can be substituted with twelve amino acids and with a truncation, but none of the substitutions reaches a number of more than 500. In contrast, arginine can be substituted with the same number of amino acids, but five of the substitutions are much more numerous than any of those found for serine or leucine.

### 2.5. Landscape of Amino Acids Introduced by Mutations Is Manifestly Dominated by Those Originating from Arginine Substitutions

Distribution of numbers of amino acids obtained in substitutions show that markedly more cysteine, histidine, and glutamine arise from substitutions of arginine than of any other amino acid (Figure 5). Additionally, virtually all newly introduced tryptophan residues originate from substitutions of arginine.

### 2.6. Validation of the CCLE Data with the Cosmic Database Confirmed Net Gains of Cysteine, Histidine and Tryptophan and Net Loss of Arginine in the Analyzed Subset of the Proteome; Arginine Was Also Found to Have the Smallest Ratio of Silent to Coding Mutations

We extracted data on single nucleotide substitutions in the same set of genes from the COSMIC database (http://cancer.sanger.ac.uk). It contains information on somatic mutations in clinical tumor samples across a wide variety of cancer types and tissue types [5]. The obtained set of data comprised circa 18,000 samples across 36 tissue types with about 812,000 observations in total. Of these, missense mutations were the biggest group with circa 569,000 events, followed by silent mutations (circa 197,000) and nonsense mutations (circa 46,000).

Our analysis of nucleotide changes showed that in total the GC pair is mutated in 78.5% of all observations, and the AT pair is mutated in 21.5% (Figure S1). The difference between the COSMIC data and the CCLE data may be explained by the absence of data on silent mutations in the CCLE database.

**Figure 6:**
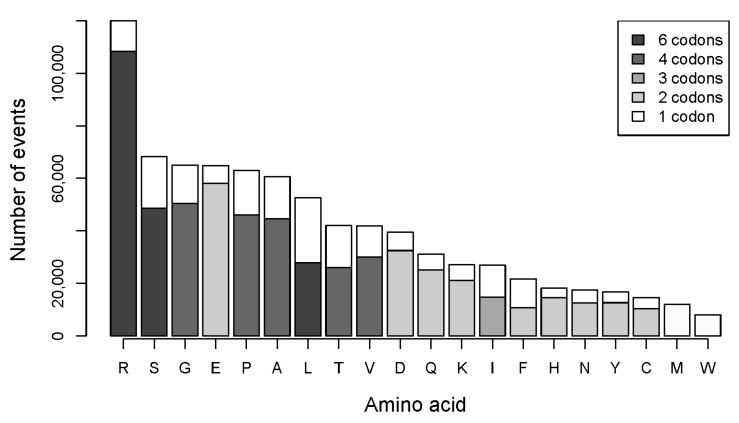
Distribution of coding and silent substitutions in the analyzed proteins for each amino acid in the Catalogue of Somatic Mutations in Cancer (COSMIC) database. The number of silent substitutions is shown in white; the number of coding substitutions is marked according to the number of codons that code for each amino acid.

Our analysis of silent mutations that arise from single nucleotide substitutions in the COSMIC database showed that arginine remains the most frequently mutated amino acid (Figure 6). Furthermore, its ratio of silent to coding substitutions is 0.11, which is the smallest value among all amino acids. The greatest values of the silent/coding ratio are observed in phenylalanine (F) and leucine (1.03 and 0.89, respectively).

We analyzed amino acid substitutions that result from single nucleotide substitutions in the studied set of genes in the COSMIC database. Similar to the CCLE data (Figure 2C), the rate of loss, calculated as the ratio of the number of the substitution events for the amino acid per 1000 samples in the COSMIC database to its amount in the 2164 proteins, is significantly different among amino acids (chi-squared = 119,410; *p* < 2.2 × 10^−16^). The rate of loss for arginine differs significantly in the chi-square test from all other amino acids and is 2.4 times higher than for the next amino acid glutamate (Figure S2) and 6.6 times higher than that for leucine that has the smallest rate of loss among all the amino acids. The rates of loss of the other amino acids are more equally distributed. Similarly to the CCLE results (Figure 2D), the most important net gains are truncation, cysteine, and histidine, the biggest loss pertains to arginine (Figure S3). The distribution of lost amino acids does not basically differ from that in the CCLE. The biggest difference in distributions of the gained amino acids between the two databases is the ranking of lysine (K) which is the fourth most gained in the COSMIC database (Figure S3) but the thirteenth in the CCLE data (Figure 3A).

Similarly to the results obtained in the analysis of the CCLE database, the mutations in cancer most avidly introduce gains of cysteine, histidine, and tryptophan residues (Figure S4) (chi square of differences in the group of the gained amino acids is 71,649, *p* < 2.2 × 10^−16^). The gains are accompanied by a prominent net loss of arginine (chi square in the group of the lost amino acids is 90,231, *p* < 2.2 × 10^−16^).

Thus, our analysis of the two databases suggests that mutagenesis in cancer causes introduction of additional residues of cysteine, histidine, and tryptophan in the proteome together with a loss of arginine.

### 2.7. The GC Pair of Nucleotides in Codons Coding for Arginine Is More Frequently Targeted in Single Nucleotide Substitutions Compared to That for Other Amino Acids. Relationship of Mutation Frequency and Codon Usage Demonstrates an Extraordinarily High Rate for Four of Six Codons of Arginine Compared to Two Other Codons of Arginine and to All Codons of Other Amino Acids

The higher oxidation potential of guanine and the high deamination ability of cytosine cause the higher mutation rate in the GC pair of nucleotides compared to that of the AT pair (Figure 1). We investigated whether the GC content in the codon composition of each amino acid is related to its rate of substitutions in proteins. We calculated the numbers of GC and AT pairs in the codons coding for each amino acid in the analyzed subset of genes from its codon composition (Table S1), codon frequency [8] and the number of amino acid residues in the subset of proteins (Figure 2A). For instance, for aspartate (D) there are two codons GAT and GAC, with frequencies 0.46 and 0.54, respectively. Thus, the frequency of occurrence of the GC pair in the codons coding for aspartate in the analyzed subset of genes is 1.54, and that of the AT pair is 1.46, totaling the sum of three for each amino acid. The obtained frequencies are multiplied by the number of aspartate residues in the 2164 proteins.

**Figure 7:**
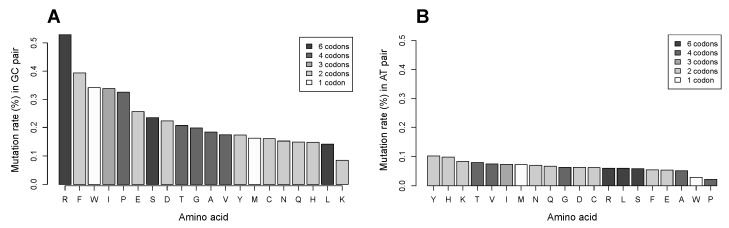
Mutation rates in the GC (**A**) and AT (**B**) pairs of nucleotides for each amino acid. The rates were calculated as ratios of single nucleotide substitutions in the GC and AT pairs (both coding and silent substitutions included) for each amino acid in the COSMIC database to the numbers of the GC and AT pairs coding for the amino acid in the analyzed subset of genes.

We extracted data on single nucleotide substitutions in the GC and AT pairs resulting in both coding and silent substitutions for each amino acid in the analyzed subset of genes from the COSMIC database. We calculated ratios of substitutions in the GC and AT pairs of nucleotides to the numbers of the GC and AT pairs, respectively. We found a strikingly uneven distribution of substitutions in the GC pair (Figure 7A), with by far the highest mutation rate in codons for arginine and lowest in those for lysine. The distribution of rates of substitutions in the AT pair is much more moderate (Figure 7B), with the highest rate in codons of tyrosine (Y) and the smallest in those of proline being comparable in magnitude. Thus, the observed high rate of substitutions of arginine in the cancer cell proteome cannot only be attributed to the GC content in the composition of its codons.

We examined the relationship of mutation frequency for each codon and codon usage for all amino acid substitutions in the analyzed data in the CCLE database. Codon usage frequency is not the same for all codons of an amino acid coded for by more than one codon, with some codons being used remarkably seldom. We calculated rates of nonsynonymous mutations for each codon using existing data on codon frequency [8] and the data on the amino acid content of the 2164 proteins (Figure 2A).

**Figure 8:**
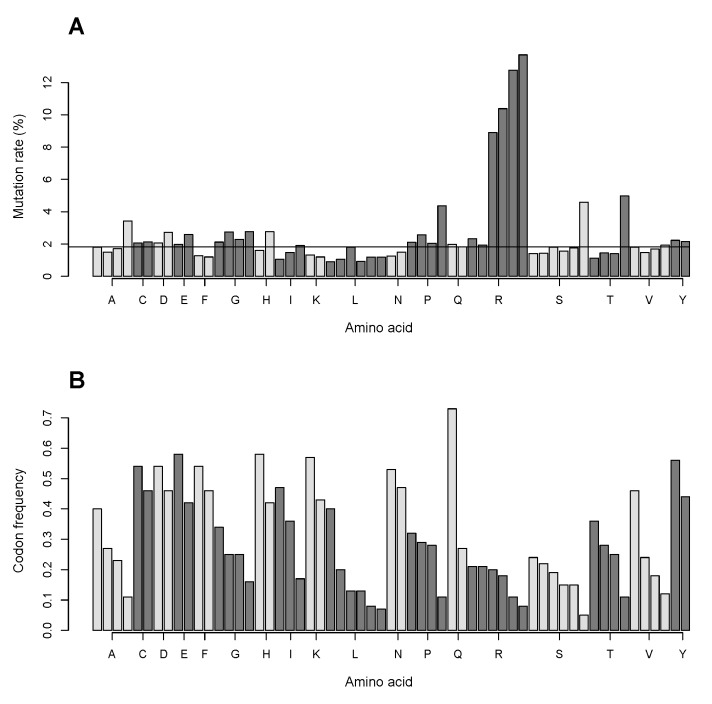
Mutation rates for codons that represent each amino acid. (**A**) The mutation rates were calculated as ratios of mutation events in the codon in the CCLE data to the number of the codon in the analyzed genes determined from the data on the codon frequencies in the human proteome [8] . The mutation rate bars for each codon are positioned according to the respective frequencies of the codons in Graph B. (**B**) Codon frequencies are ranked in the descending order for codons of each amino acid (Table S1). In both graphs, bars pertaining to each amino acid are alternately represented in dark grey and light grey for convenience of perception.

We found that for serine, threonine, proline, and to a smaller extent, alanine the mutation rate is markedly higher in low-usage codons (Figure 8). However, the low-usage codon effect is virtually nonexistent in leucine, where the smallest codon frequencies are 0.08 and 0.07. The possible effect of low-usage codon for arginine (CGT; frequency 0.08) by far exceeds that for serine (TCG; frequency 0.05). The increased mutation rate in arginine is not limited to the low-usage codon but is rather characteristic for four of its six codons, with two of the highly mutated codons having codon frequencies similar to those of the two low mutated codons (Figure 8).

The combined top amino acid transformation data for the mutations in the four codons of arginine in the CCLE database yield histidine (1414 transformations), cysteine (1267), glutamine (1267), tryptophan (943), and nonsense (*) (686). It makes 52.6% of the total number of histidine obtained due to mutations, as well as 51.6% of cysteine, 62.8% of glutamine, 77.4% of tryptophan, 23.2% of nonsense mutations in the whole database, contributing almost solely to the net gain of cysteine and histidine (Figure 2D) and exclusively to the gain of tryptophan. Mutations in the low-usage codons of alanine (A), proline, threonine (T), and serine predominantly give rise to valine (304 events), leucine (373), methionine (463), and leucine (280), respectively.

The preference for certain codons over others is highly related to the presence of the CG sequence in the codon. Of all codons, the sequence (Table S1) is present only in those that have high and very high mutation rates, namely in the low-usage codons of alanine, proline, threonine, and serine (GCG (0.11), CCG (0.11), ACG (0.11), TCG (0.05), respectively, codon frequency is denoted in brackets) and in four codons of arginine (CGG (0.20), CGC (0.18), CGA (0.11), CGT (0.08)).

### 2.8. All Tissue Types in Cancer Undergo Loss of Arginine and Gain of Cysteine, and Most Tissue Types Gain Histidine and Tryptophan in Amino Acid Substitutions in the Analyzed Subset of Proteins. The Patterns of a Marked Loss of Arginine and High Gains of Cysteine and Histidine as Well as Uniform Gains of Some Amino Acids and Losses of the Others Are Universal for the Total Cancer Proteome

**Figure 9:**
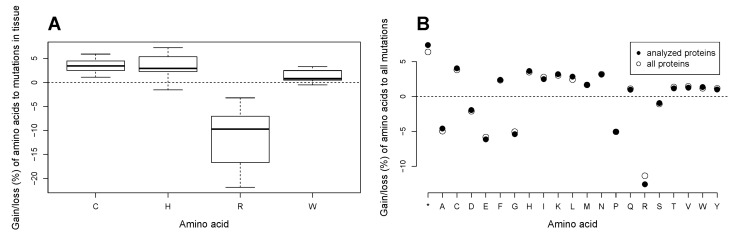
(**A**) Rates of gain/loss of cysteine, histidine, arginine, and tryptophan in the analyzed proteome subset in 23 tissue types in the COSMIC database. The data are calculated as percentages of net gains or losses of the amino acids to the total number of amino acid substitutions in the analyzed proteome subset. (**B**) Comparison of gain/loss of amino acids between the analyzed proteome subset and the total set of proteins in the COSMIC database. The rates of gains/losses of amino acids are calculated as ratios of the net gain or loss of each amino acid to the total number of substitutions in the dataset in both cases.

We further investigated whether there are tissue-specific patterns in gains and losses of cysteine, histidine, arginine, and tryptophan in the analyzed subset of proteins with the data in COSMIC. For the analysis, we extracted data on amino acid substitutions in those tissue types where the number of tumor samples was more than one hundred. The data yielded 23 tissue types (Table S2) with the number of tumor samples from 166 (adrenal gland) to 2109 (hematopoietic and lymphoid tissue). The mean numbers of nonsynonymous amino acid substitutions per sample ranged from 4.2 (autonomic ganglia) to 113.4 (skin) to 149.4 (endometrium), while the median numbers ranged from 3 (adrenal gland and autonomic ganglia) to 45 (skin). To account for the varying numbers of tumor samples, the mutation rates for the four amino acids were calculated as percentages of the net gain or loss of the amino acid to the total number of substitutions in all tumor samples pertaining to the tissue type. We found that arginine is consistently lost in all tissues (Figure 9A), in a very wide range from −3.2% in adrenal gland to −20.7% in large intestine to −21.8% in the central nervous system (Table S3). Similarly, cysteine is acquired in mutations in all tissues, with the lowest gain in the upper aerodigestive tract and highest gains in the central nervous system, stomach, and large intestine. While histidine is gained in almost all tissue types, with the highest percentages of gain in the central nervous system, stomach and large intestine, it is lost in skin cancers. Likewise, tryptophan is only lost in skin cancers, but gained in all others, with the highest gains in the large intestine, pancreas, bone, and stomach. To account for a possible confounding effect of the high numbers of arginine substitutions in p53, we also performed the analysis with exclusion of data on p53, and obtained similar results.

While the changes in the protein composition were unidirectional for almost all tissue types, their percentages of gains or losses differed in magnitude. Indeed, an analysis of variance (ANOVA) showed significant differences between tissue types in gains/losses of each of the four amino acids. A posthoc analysis with Holm correction for multiple testing revealed that cancers of endometrium, large intestine, stomach, skin, and lung differed from those of the other tissues in terms of their respective net gains and losses of the amino acids. The findings may to some extent be attributed to the high mean numbers of mutations in the tissues (Figure S5). Nevertheless, the obtained results may also reflect some underlying differences between tissue types thus suggesting possible existence of tissue-specific amino acid gain/loss signatures. In skin cancers, for instance, cysteine is avidly gained and arginine is lost, as in the other tissue types. Yet, skin cancers lose histidine and tryptophan while the other tissue types gain the amino acids (Figure S5).

The current analysis was performed on a subset of circa two thousand proteins that approximately amounts to one tenth of the human proteome. In order to see whether the observed phenomena of gains of some amino acids and losses of the others are pertinent to all proteins in the cancer cell, we analyzed data on all nonsynonymous amino acid substitutions that arise from single nucleotide substitutions in the COSMIC database. For this, we examined data on approximately three million amino acid substitutions in circa 29,000 proteins in more than 18,000 tumor samples belonging to 36 tissue types. For both the subset of analyzed proteins and the dataset containing all proteins, rates of gains/losses of amino acids were calculated. We found that gains of truncated proteins (*), cysteine (C), phenylalanine (F), histidine (H), isoleucine (I), lysine (K), leucine (L), methionine (M), asparagine (N), glutamine (Q), threonine (T), valine (V), tryptophan (W), tyrosine (Y) as well as losses of alanine (A), aspartate (D), glutamate (E), glycine (G), proline (P), arginine (R), and serine (S) are universally characteristic for the proteome of the cancer cell in all tissue types combined (Figure 9B). In the both sets of proteins, the biggest percentages of gain pertain to cysteine and histidine, and the by far greatest losses are found for arginine. No difference in percentages of gain/loss of amino acids between the data on the analyzed proteome subset and the data on all COSMIC proteins was found (paired *t*-test, *p* = 0.99997). However, the graph indicates that arginine undergoes a slightly greater loss in the analyzed subset of proteins than in the total proteome. As the CCLE database predominantly lists well-established cancer related genes, the slightly greater loss of arginine may signify the importance of the amino acid in the functioning of proteins implicated in cancer. In addition, our above findings suggest that different cancer types may have distinct signatures of gain/loss of amino acids.

### 2.9. Loss of Arginine Is Prominent in Several Tumor Suppressor Proteins

We extracted data on substitutions of arginine in individual proteins from the CCLE database. A number of 1526 proteins had at least one event of substituted arginine per 782 CCLs, with proteins coded by *TTN* (295 events in 203 CCLs), *TP53* (188, 183 CCLs), *OBSCN* (55, 45 respectively), *ADAMTSL3* (37, 37), *LRP1B* (36, 36), *LRP2* (36, 31), *HERC2* (36, 35) being most often affected by arginine substitutions. We calculated the ratio of events of substitutions of arginine in the protein across all cancer cell lines in the CCLE database to the total number of arginine residues in the protein molecule. The top twelve proteins with the highest arginine substitution ratio are shown in Table 1. The high ratio of substitutions of arginine to the number of arginine residues in the proteins may indicate importance of arginine for their functioning.

We analyzed frequencies of shared positions of substitutions of arginine in individual proteins in the CCLE database (Table S4) and in the COSMIC database (Table S5). In both databases, the top most frequent positions are dominated by mutations of p53 protein. Apart from *TP53*, the proteins are coded by genes *FBXW7*, *IDH1*, *CDKN2A*, *PTEN*, *GNAS*, *SMAD4*, *PDE4DIP*, *APC* in the COSMIC database.

We used the FATHMM (Functional Analysis through Hidden Markov Models) score [9] data listed in the COSMIC database to assess possible deleterious effects of arginine substitutions. We found that of thirteen possible substitutions of arginine, the highest mean FATHMM scores are yielded for truncation and substitutions with isoleucine, histidine, cysteine, glutamine, and tryptophan (0.83, 0.79, 0.78, 0.77, and 0.76, respectively) that may implicate the possible role of the substitutions in cancer progression. We also calculated the mean FATHMM scores for the top frequently shared substitutions of arginine in the COSMIC database (Table S5). Thirty-seven positions of frequent arginine loss in proteins in cancer out of the top 56 yield a FATHMM score of more than 0.9 suggesting high pathogenicity of the loss of arginine in the sites. Only six frequent positions of arginine loss are predicted to be neutral, with mean FATHMM scores less than 0.1 (Table S5).

## 3. Discussion

In coding missense mutations, a substitution of a nucleotide ultimately results in a change of an amino acid that alters properties of the protein to various extents depending on the difference in chemical properties of the lost amino acid and the gained one and the effect it causes on the structure of the protein. However, little attention has been drawn to global effects of the amino acid substitutions on the proteome level. In the current study, we analyzed effects of single nucleotide substitutions in genes that code for 2164 cellular proteins, on the amino acid composition of the subset of the proteome. The mutation data were extracted from the Cancer Cell Line Encyclopedia [4] that contains information of genomic and proteomic alterations in 782 cancer cell lines. The analysis was validated and expanded with mutation information for the same set of genes in COSMIC (v78), the Catalogue of Somatic Mutations in Cancer [5] in circa 18,000 tumor samples. The findings were further confirmed with an analysis of all amino acid alterations that result from single nucleotide substitutions in approximately 29,000 unique proteins in the database.

Our analysis of the two big databases of mutations in cancer shows that coding mutations introduce most marked net gains of cysteine, histidine, and a relative gain of tryptophan in the proteins in the cancer cell proteome across multiple tissue types, and the gains are almost solely attributed to a loss of arginine. Other amino acids are also uniformly gained or lost across the cancer cell proteome albeit at smaller rates (Figure 9B).

Arginine has highly unusual properties in mutations in cancer. While we and others [3] showed that arginine is most frequently lost in mutations in cancer, and substitutions of arginine belong to a few exceptionally numerous amino acid substitutions, here we use a consistent subset of the proteome to demonstrate that not only is arginine lost in numbers disproportionate to its content in the proteins, but the proteome subset also undergoes a significant net loss of arginine and its replacement with cysteine, histidine, and tryptophan in cancer cells. We analyzed a possible effect of composition of codons coding for arginine on its extraordinary predisposition to mutations. We showed that the GC pair in codons coding for arginine is targeted at a much higher rate than that in other amino acids. Arginine is in place nine for its content in the analyzed set of proteins (Figure 2A) but is coded for with six codons like the most abundant amino acids leucine and serine. We found that for four of its six codons the mutation rate is markedly higher than for all codons coding for other amino acids (Figure 8). While for serine, threonine, proline, and to a smaller extent, alanine the mutation rate is substantially higher in low-usage codons, the increased mutation rate in arginine is not limited to its least frequent codon. Low usage codons also have a high probability of being rare, i.e., being decoded with low-abundant tRNA [10]. Rare codons are often found in late/slowly expressed genes [11], and the high mutation rate in them is in line with the current notion that many passenger mutations occur in genes that replicate late in the cell cycle and/or have low expression levels [1]. However, the low-usage codon effect is virtually nonexistent in leucine, where the smallest codon frequencies are 0.08 and 0.07. The possible effect of low-usage codon for arginine (CGT; frequency 0.08) by far exceeds that for serine (TCG; frequency 0.05). Two of the four arginine codons that display high mutation rates in our study have high codon frequencies (Figure 8). While the codon frequency data are derived from the total human proteome and might not exactly match the codon frequency for genes encoding the analyzed set of proteins, it is unlikely that any possible mismatch only pertains to arginine and not to other amino acids. Transformations of these four codons result in more than half of all substitutions introducing cysteine and histidine residues, and more than three quarters of all tryptophan residues, as well as about a quarter of all truncation events. As such, the mutations in the four arginine codons are almost solely responsible for the net gains of cysteine and histidine (Figure 2D) and exclusively responsible for the net gain of tryptophan.

Additionally, a higher number of codons for a same amino acid can be expected to protect the amino acid against mutations by increasing the number of possible transformations that lead to synonymous substitutions. Surprisingly, our analysis suggests that having six codons does not protect arginine from being most frequently lost in mutations in cancer. To the contrary, the ratio of silent to coding substitutions of arginine is smallest among all amino acids, being ten times smaller than that for phenylalanine and eight times smaller than that for leucine.

Remarkably, the persistent loss of arginine makes somatic evolution in cancer different from evolution of the proteome that accompanies increasing complexity of organisms, where cysteine, methionine, histidine, serine, and phenylalanine are accrued in proteins while proline, alanine, glutamate, and glycine are consistently lost [12].

Here we show that exceptionally high mutation rates in four codons of arginine and high mutation rates in the low-usage codons of alanine, proline, threonine, and serine (Figure 8) are associated with the presence of the CG sequence in the codon composition. None of the codons coding for the other amino acids contains the CG sequence. The possible cause for the high and very high mutation rates in these codons and not in the others can be the deamination of methylated cytosines in CpG sequences where the high deamination ability of cytosine promotes its conversion to thymine [6]. The pattern may reflect the aberrant DNA methylation in cancer [13,14]. As such, loss of arginine in proteins due to mutations may provide information about methylation hot spots in cancer.

However, the mutation rate for the four codons of arginine is still much higher than that for the other four amino acids that have the CG sequence in their codons. One possible reason for arginine being disproportionately lost in mutations in cancer is its important role in those tumor suppressor proteins that bind nucleic acids or nucleotides, where a positively charged arginine residue provides bonding of a negatively charged phosphate group. An important example of such a loss of function is the p53 protein, where these mutations occur within the DNA binding domain and alter the DNA binding properties of p53 and its ability to act as a tumor barrier [15]. For those proteins, high frequency of the same individual positions of arginine substitution is observed that may indicate that this amino acid is important for maintenance of critical protein functions. While p53 has an exceptionally high ratio of events of arginine substitution to the total number of arginine in the molecule, it is only responsible for 2.7% of all arginine substitutions in the CCLE database. Nevertheless, in most other proteins loss of arginine is evenly distributed. For instance, the number of arginine substitutions with five or more events in the same position in an individual protein is only 7.3% of the total number of substitutions of arginine in the CCLE database.

Another reason for the very high mutation rates in the four codons of arginine compared to the CG containing codons of alanine, proline, threonine, and serine is the possible adaptive role of the amino acid residues that are introduced into proteins in place of arginine. Сhemical properties and functions of the gained amino acids suggest that the changes may increase the total antioxidant and metal binding capacity of the proteome, possibly counteracting aggravated oxidation stress that is an important feature of the cancer cell. Given the high count of passenger mutations that is common for some cancers and/or high copy numbers of the protein molecule that carries the substitution, it might be supposed that the sum of the changes may produce a distinct shift in the proteome chemistry in cancer.

Free thiol groups in cysteine residues in cell proteins are strong reducing agents that can neutralize excessive reactive oxygen species (ROS) and participate in redox signaling. The thiol group of cysteine can also bind metal ions with high affinity. Due to its specialized functions and high chemical reactivity cysteine is mostly found in functional sites of proteins [16]. Cysteine is believed to be a later addition to the genetic code [17], its representation appears to correlate positively with the complexity of the organism [18] and its accumulation is ongoing in present day organisms [12]. Due to its high functionality, cysteine has an extreme conservation pattern, being either highly conserved or poorly conserved [16] following its introduction into proteins. Tryptophan follows the same conservation patterns as cysteine albeit to a smaller extent [16]. Histidine also binds metal ions. Like cysteine, it can coordinate redox active iron ions and prevent them from taking part in the Fenton reaction that generates deleterious oxidants. Additionally, histidine also participates in acid-base catalysis, and its introduction may lead to the formation of new catalytic sites.

The de novo introduction of the three amino acids with highly specialized functions in somatic evolution in cancer may possibly contribute to the fitness of the cancer cell. However, for the cancer cell, an increased de novo incorporation of cysteine, histidine or tryptophan into proteins places additional demands on its metabolism and may make some cancers with high number of net gain of these or other amino acids in mutations sensitive to deprivation of the nutrients. It has been recently shown that cutting out certain amino acids from the diet of mice slows tumor growth and prolongs survival [19]. The present study suggests that quantifying the changes of the proteome composition in cancer mutations can provide a rational basis for dietary interventions aimed at aiding cancer treatment.

It remains to be investigated whether the gains of cysteine, histidine, and tryptophan play a role in cancer progression. In addition, it is unclear as to what extent the net loss of arginine in multiple proteins affects cancer cell metabolism. Furthermore, we show here that gains of cysteine and histidine at the expense of the predominant loss of arginine are universally most prominent across multiple tissue types; we also show that other amino acids are consistently gained or lost in the proteome of the cancer cell (Figure 9B). Magnitudes and combinations of the amino acid gain/loss patterns in individual tissue types deserve further investigation as they may suggest existence of tissue-specific signatures of proteome alterations in cancer and thus reveal metabolic dependencies of individual cancers.

## 4. Materials and Methods

We extracted information on nucleotide substitutions and resulting amino acid substitutions from the CCLE database (https://portals.broadinstitute.org/ccle) for protein-coding genes for which Swiss-Prot protein IDs were listed in the database. Similarly, information on single nucleotide substitutions and resulting amino acid substitutions in the same set of protein coding genes was extracted from the COSMIC (v78) database (http://cancer.sanger.ac.uk). Protein sequences were downloaded from the UniProt database (http://www.uniprot.org) and the content of each amino acid in the proteins was calculated. Statistical analysis was performed in R version 3.3.1 [20] using RStudio version 0.99.893 [21].

## Figures and Tables

**Table 1:** Proteins with the highest ratio of events of substitutions of arginine in the CCLE database to the total number of arginine residues in the protein molecule.

Gene Coding for the Protein	Number of Arginine Substitution Events in the Protein	Total Number of Substitutions in the Protein in the CCLE Database	Number of Arginine Residues in the Protein	Number of Amino Acids in the Protein	Ratio of Substituted Arginines to Total Number of Mutations	Ratio of Substituted Arginines to Number of Arginine Residues	Ratio of all Non-Arginine Substitutions to Total number of Non-Arginine Amino acids
*TP53*	188	458	26	393	0.41	7.23	0.74
*PTEN*	22	74	20	403	0.30	1.10	0.14
*FBXW7*	26	44	43	707	0.59	0.60	0.03
*CASP8*	11	27	26	479	0.41	0.42	0.04
*STK4*	10	14	24	487	0.71	0.42	0.01
*TNNI3K*	13	66	36	835	0.20	0.36	0.07
*ADAMTSL3*	37	101	106	1691	0.37	0.35	0.04
*GNAS*	10	54	29	394	0.19	0.34	0.12
*ZNF423*	14	51	47	1284	0.27	0.30	0.03
*HIP1*	17	33	59	1037	0.52	0.29	0.02
*RB1*	13	58	46	928	0.22	0.28	0.05
*CDK18*	10	18	36	472	0.56	0.28	0.02

## Supplementary Materials

The following are available online at http://www.mdpi.com/2218-273X/7/3/49/s1, Figure S1: Difference in numbers of mutations between the GC and AT pairs of nucleotides in the COSMIC database, Figure S2: Rate of loss for each amino acid in the COSMIC database, Figure S3: Frequencies of gain/loss of amino acids due to coding substitutions in the analyzed proteome subset per 1000 samples in the COSMIC database, Figure S4: Rate of gain/loss for each amino acid in the COSMIC database, Figure S5: Net gain/loss of cysteine, histidine, arginine and tryptophan in the analyzed subset of the proteome per sample in 23 tissues in the COSMIC database, Table S1: Codon frequencies in the human proteome, Table S2: Statistics of coding mutations per tissue in the analyzed subset of the proteome in the COSMIC database, Table S3: Gain/loss of the four amino acids calculated as percentage of the net gain/loss to the total number of mutations per tissue in the analyzed subset of the proteome in the COSMIC database, Table S4: Top frequencies of shared positions of substitutions of arginine in individual proteins in the CCLE database, Table S5: Top frequencies of shared positions of substitutions of arginine in individual proteins in the COSMIC database.
