# Supplementary material for: Mutations in Cancer Cause Gain of Cysteine, Histidine, and Tryptophan at the Expense of a Net Loss of Arginine on the Proteome Level
Source: Biomolecules. 2017 Jul 3;7(3):49. doi: 10.3390/biom7030049 (PMC5618230; doi:10.3390/biom7030049)
Supplement: Supplementary file 1 [file biomolecules-07-00049-s001.pdf]

SUPPORTING INFORMATION

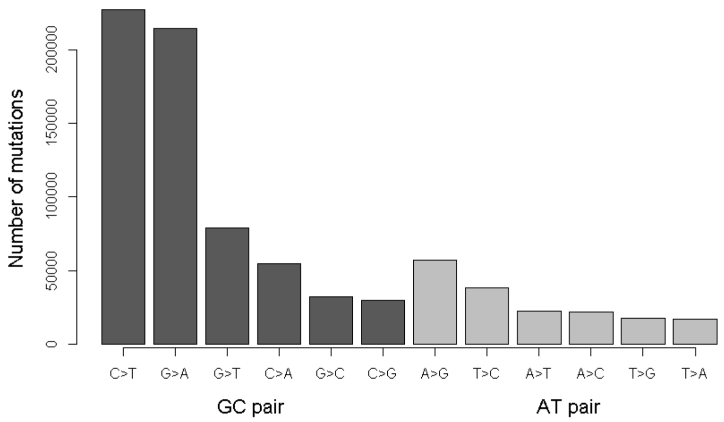

Figure S1. Difference in numbers of mutations between the GC and AT pairs of nucleotides in the COSMIC database.

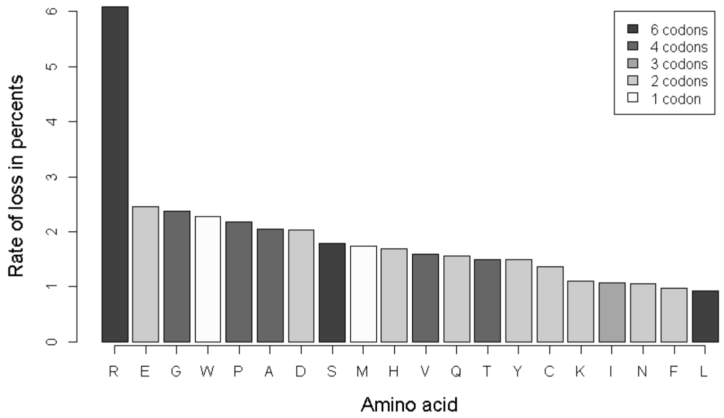

Figure S2. Rate of loss for each amino acid. It is calculated as the ratio of the number of the substitution events for the amino acid per 1000 samples in the COSMIC database to its amount in the 2164 proteins.

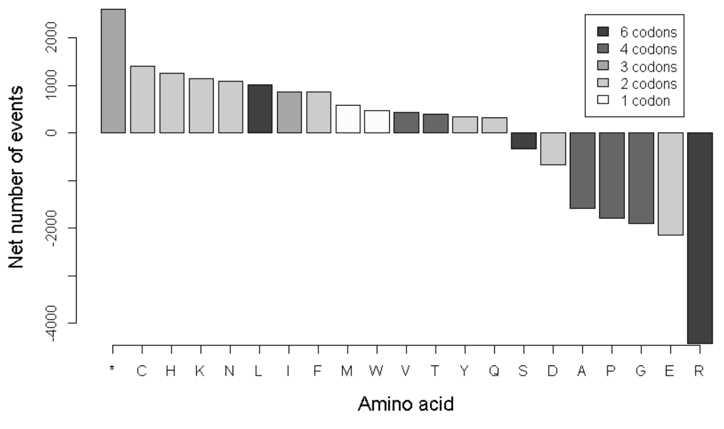

Figure S3. Frequencies of gain/loss of amino acids due to coding substitutions in the analyzed proteome subset per 1000 samples in the COSMIC database.

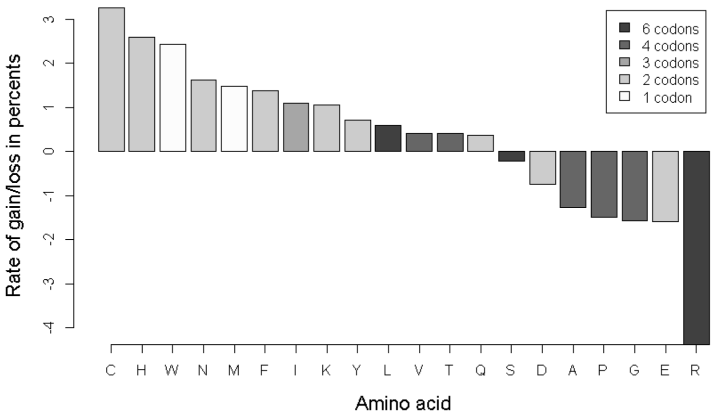

Figure S4. Rate of gain/loss for each amino acid. The rate was calculated as the ratio of the net frequencies of its gain/loss in coding substitutions to its amount in the analyzed proteome subset per 1000 samples in the COSMIC database.

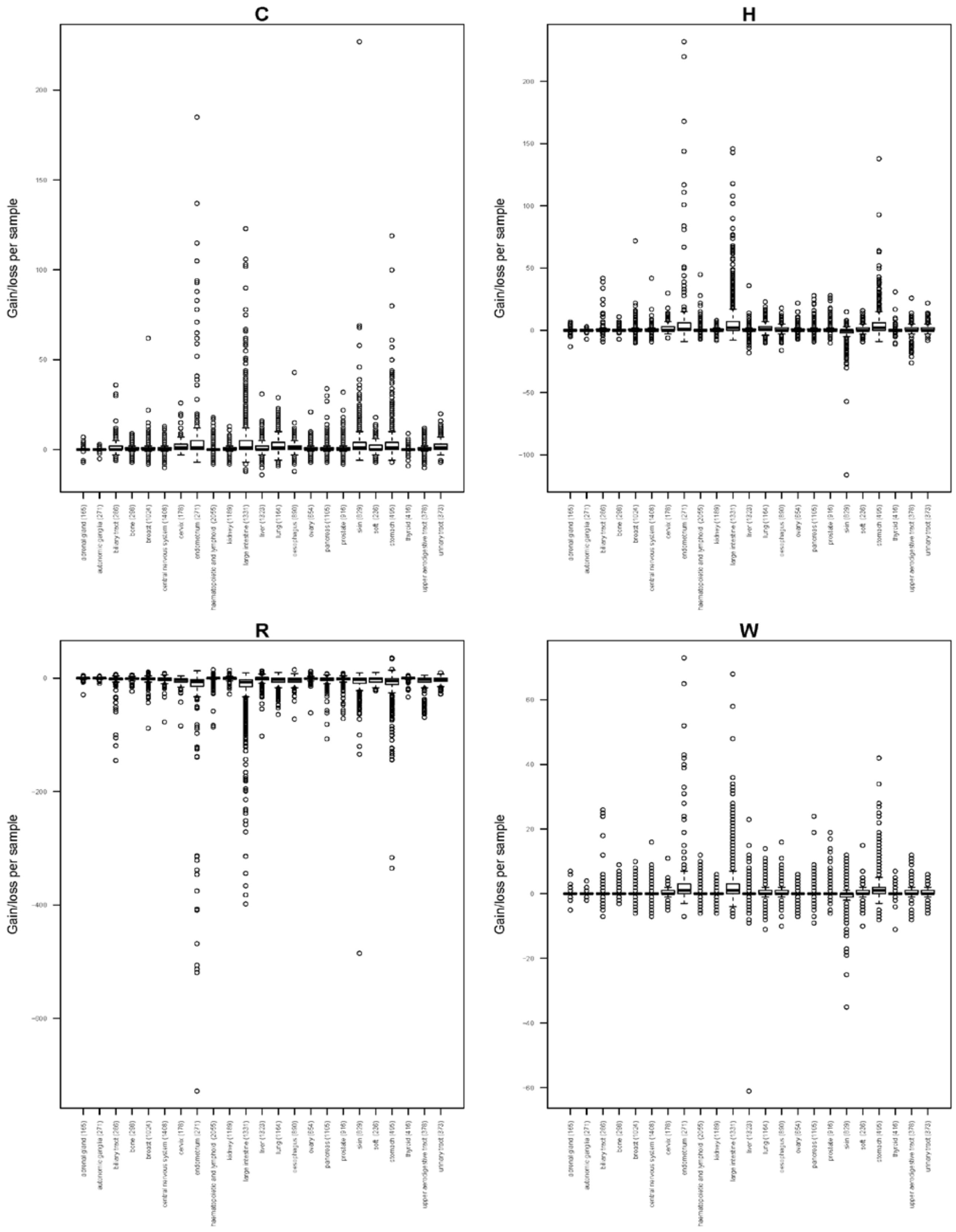

Figure S5. Net gain/loss of cysteine, histidine, arginine and tryptophan in the analyzed subset of the proteome per sample in 23 tissues in the COSMIC database.

Table S1. Codon frequencies in the human proteome (http://www.kazusa.or.jp).

Codone	Amino acid (name)	Amino acid (letter)	Frequency
GCC	Ala	A	0.40
GCT	Ala	A	0.27
GCA	Ala	A	0.23
GCG	Ala	A	0.11
AGA	Arg	R	0.21
AGG	Arg	R	0.21
CGG	Arg	R	0.20
CGC	Arg	R	0.18
CGA	Arg	R	0.11
AAC	Asn	N	0.53
AAT	Asn	N	0.47
GAC	Asp	D	0.54
GAT	Asp	D	0.46
TGC	Cys	C	0.54
TGT	Cys	C	0.46
CAG	Gln	Q	0.73
CAA	Gln	Q	0.27
GAG	Glu	E	0.58
GAA	Glu	E	0.42
GGC	Gly	G	0.34
GGA	Gly	G	0.25
GGG	Gly	G	0.25
GGT	Gly	G	0.16
CAC	His	H	0.58
CAT	His	H	0.42
ATC	Ile	I	0.47
ATT	Ile	I	0.36
ATA	Ile	I	0.17
CTG	Leu	L	0.40
CTC	Leu	L	0.20
TTG	Leu	L	0.13
CTT	Leu	L	0.13
TTA	Leu	L	0.08
CTA	Leu	L	0.07
AAG	Lys	K	0.57
AAA	Lys	K	0.43
ATG	Met	M	1.00
TTC	Phe	F	0.54
TTT	Phe	F	0.46
CCC	Pro	P	0.32
CCT	Pro	P	0.29
CCA	Pro	P	0.28
CCG	Pro	P	0.11
AGC	Ser	S	0.24
TCC	Ser	S	0.22
TCT	Ser	S	0.19
TCA	Ser	S	0.15
AGT	Ser	S	0.15
TCG	Ser	S	0.05
ACC	Thr	T	0.36
ACA	Thr	T	0.28
ACT	Thr	T	0.25
ACG	Thr	T	0.11

TGG	Trp	W	1.00
TAC	Tyr	Y	0.56
TAT	Tyr	Y	0.44
GTG	Val	V	0.46
GTC	Val	V	0.24
GTT	Val	V	0.18
GTA	Val	V	0.12
TGA	STOP		0.47
TAA	STOP		0.30
TAG	STOP		0.24

Table S2. Statistics of coding mutations per tissue in the analyzed subset of the proteome in the COSMIC database.

Tissue	Total number of mutations	Number of samples	Mean number of mutations	Median number of mutations
Adrenal gland	1247	165	7.56	3
Autonomic ganglia	1212	271	4.47	3
Biliary tract	6517	286	22.79	10
Bone	3482	298	11.68	6
Breast	20689	1024	20.2	12
Central nervous system	14967	1408	10.63	8
Cervix	8016	178	45.03	24
Endometrium	40640	271	149.96	24
Haematopoietic and lymphoid tissue	18150	2055	8.83	4
Kidney	16182	1189	13.61	12
Large intestine	115420	1331	86.72	33
Liver	36895	1323	27.89	21
Lung	66844	1164	57.43	41
Oesophagus	26965	890	30.3	27
Ovary	9144	654	13.98	11
Pancreas	17945	1105	16.24	13
Prostate	13163	916	14.37	9
Skin	94007	829	113.4	45
Soft tissue	12246	236	51.89	35.5
Stomach	39426	495	79.65	30
Thyroid	5290	416	12.72	3.5
Upper aerodigestive tract	24659	378	65.24	19
Urinary tract	14306	373	38.35	26

Table S3. Gain/loss of the four amino acids calculated as percentage of the net gain/loss to the total number of mutations per tissue in the analyzed subset of the proteome in the COSMIC database.

Tissue	Cysteine	Histidine	Arginine	Tryptophan
Adrenal gland	2.486	1.443	-3.208	0.722
Autonomic ganglia	1.568	1.98	-9.158	0.743
Biliary tract	5.616	5.048	-18.95	2.378
Bone	3.188	2.814	-12.665	2.843

Breast	3.76	2.77	-9.246	1.276
Central nervous system	5.232	6.588	-21.828	2.479
Cervix	4.229	3.942	-11.115	0.699
Endometrium	4.924	5.652	-21.134	2.483
Haematopoietic and lymphoid tissue	3.025	4.452	-11.361	1.355
Kidney	2.095	2.855	-4.616	0.198
Large intestine	5.604	7.266	-20.715	3.09
Liver	2.499	1.184	-5.014	0.426
Lung	3.899	2.76	-7.099	0.298
Oesophagus	3.456	3.772	-12.902	1.687
Ovary	2.297	3.937	-7.677	0.087
Pancreas	4.508	6.353	-17.409	2.903
Prostate	4.102	6.184	-15.961	3.297
Skin	3.167	-1.554	-6.956	-0.493
Soft tissue	2.768	2.556	-6.304	0.612
Stomach	5.91	7.084	-17.618	2.625
Thyroid	1.285	1.89	-4.159	0.397
Upper aerodigestive tract	1.103	0.75	-9.721	0.783
Urinary tract	4.467	2.936	-8.514	0.517

Table S4. Top frequencies of shared positions of substitutions of arginine in individual proteins in the CCLE database.

**Protein-coding gene**	**Position**	**Number of cases**
TP53	248	56
TP53	273	48
TP53	175	19
TP53	213	14
TP53	282	8
TP53	110	7
TP53	158	7
TP53	280	7
TP53	249	5
TP53	306	5
ADAMTSL3	855	27
ADAMTSL3	59	5
TTN	9741	14
TTN	21700	7
TTN	25126	7
TTN	12674	6
TTN	13564	6
CDC42BPA	1198	13
HERC2	2126	11
PTEN	130	10
PTEN	233	5
HIP1	508	10
CUBN	651	10
TRPS1	801	10
STK4	117	9
FBXW7	465	9
FBXW7	505	6

FBXW7	479	5
PAPPA	758	9
TG	1066	8
NPAT	230	7
STYK1	379	7
ATP8B1	384	7
RGS22	1108	7
FANCM	1644	7
ERBB2	143	6
CCKBR	215	6
TNK2	382	6
ROS1	2039	6
TG	2585	6
UNC13C	36	5
CASP8	68	5
CDKN2A	80	5
SCARA5	96	5
ATR	109	5
MECOM	114	5
ATP10A	244	5
CDKL4	307	5
SMAD4	361	5
TNNI3K	406	5
IRAK2	504	5
ITK	581	5
DVL1	594	5
CDK13	1366	5
APC	1450	5
UBR5	1907	5

Table S5. Top frequencies of shared positions of substitutions of arginine in individual proteins in the COSMIC database.

Protein-coding gene	Position	Number of cases	Mean FATHMM score
TP53	273	1456	0.998
TP53	248	1427	0.963
TP53	175	1312	0.997
TP53	213	744	0.989
TP53	282	618	0.987
TP53	249	435	0.459
TP53	196	424	0.988
TP53	155	311	0.963
TP53	158	302	0.997
TP53	43	282	0.986
TP53	82	282	0.998
TP53	306	243	0.947
TP53	280	193	0.968
TP53	342	179	0.69
TP53	81	169	0.971
TP53	120	165	0.988
TP53	267	94	0.989

TP53	110	92	0.125
TP53	64	90	0.989
TP53	103	90	0.989
TP53	337	88	0.799
TP53	65	84	0.813
TP53	26	68	0.994
TP53	181	67	0.901
TP53	156	53	0.463
IDH1	132	442	0.948
FBXW7	465	186	0.769
FBXW7	505	120	0.992
FBXW7	226	92	0.77
FBXW7	385	92	0.77
FBXW7	347	81	0.975
FBXW7	479	64	0.992
FBXW7	425	62	0.952
FBXW7	266	60	0.983
FBXW7	387	52	0.981
CDKN2A	80	129	0.863
CDKN2A	58	54	0.265
PTEN	130	115	0.969
PIK3CA	88	102	0.964
GNAS	844	92	0.961
GNAS	201	91	0.962
SMAD4	361	74	0.972
PDE4DIP	622	74	0.068
PDE4DIP	681	72	0.042
PDE4DIP	25	70	0.088
PDE4DIP	2291	56	0.006
PDE4DIP	171	48	0.107
PDE4DIP	1504	46	NA
PDE4DIP	1867	46	NA
APC	1450	74	0.903
APC	876	63	0.951
CHEK2	519	52	0.492
ZAN	1922	52	0.513
USP6	69	50	0.003
MAX	60	47	0.993
NBPF12	36	46	NA
